# Barriers and Facilitators of Implementation of the Non-Hospital-Based Administration of Long-Acting Cabotegravir Plus Rilpivirine in People with HIV: Qualitative Data from the HOLA Study

**DOI:** 10.3390/v17070993

**Published:** 2025-07-16

**Authors:** Diana Hernández-Sánchez, Juan M. Leyva-Moral, Julian Olalla, Eugènia Negredo, on behalf of the HOLA Study Group

**Affiliations:** 1 Lluita Contra les Infeccions, Hospital Universitari Germans Trias i Pujol, Ctra del Canyet S/N, 08916 Badalona, Spain; enegredo@lluita.org; 2 Departamento de Medicina, Universitat Autònoma de Barcelona, 08193 Bellaterra, Spain; 3 Grup de Recerca Infemera en Vulnerabilitat i Salut, Departament d’Infermeria. Universitat Autonoma de Barcelona, 08015 Barcelona, Spain; juanmanuel.leyva@uab.cat; 4 Centro de Investigación Biomédica en Red de Epidemiología y Salud Pública (CIBERESP), Instituto de Salud Carlos III, 28029 Madrid, Spain; 5 Hospital Universitario Costa del Sol, 29603 Marbella, Málaga, Spain; julian.olalla.sspa@juntadeandalucia.es; 6 Universitat de Vic-Universitat Central de Catalunya (UVic-UCC), 08500 Vic, Spain

**Keywords:** HIV, antiretrovirals, cabotegravir + rilpivirine long acting, out-of-hospital administration, implementation, barriers and facilitators, qualitative methods

## Abstract

Long-acting (LA) antiretroviral therapies for human immunodeficiency virus (HIV), such as injectable formulations of cabotegravir and rilpivirine (CAB+RPV LA), are now available. Considering the limited data on the out-of-hospital administration of this combination, evaluating the implementation strategies needed is essential to support future clinical efforts. To gather data on barriers and facilitators of implementation for CAB+RPV LA in alternative outpatient facilities, this study used qualitative interviews informed by the Consolidated Framework for Implementation Research (CFIR), with 13 staff participating in the HOLA study (NCT06185452). Data analysis followed qualitative descriptive methods, assisted by Atlas.ti software version 22. The study adhered to the COREQ guidelines. Findings reveal five main factors to consider for implementation: operational and infrastructure adaptations, integrated management of human and organizational resources, need for coordination and follow-up, professional attitudes and work environment, and patient experience and patients’ needs perceived by professionals. This study emphasizes the comprehensive operational and infrastructure adaptations, adequate staff training, and supportive professional environment required for the successful implementation of CAB+RPV LA, while considering patients’ needs throughout the externalization process (trial registration number: NCT06643897).

## 1. Background

Long-acting (LA) antiretroviral therapies for human immunodeficiency virus (HIV), such as injectable formulations of cabotegravir and rilpivirine (CAB+RPV LA), are now available. They represent great advancements made by reducing the dosing frequency of treatment and avoiding oral formulation, enhancing convenience and quality of life for people with HIV (PWH) [1,2,3]. The CARISEL study, focused on CAB+RPV LA administration in many European centers, demonstrated high acceptability and feasibility of the regimen in hospital-based clinical settings [4,5,6,7]. However, approximately two years after the introduction of CAB+RPV LA, in Spain, CAB+RPV LA is mainly limited to hospital-based outpatient administration, as in many other countries. Evidence regarding the non-hospital-based administration of CAB+RPV LA is very limited, and only a few studies are aiming to evaluate the feasibility of the non-hospital-based administration of CAB+RPV LA, and only preliminary data are available [8,9,10,11].

The ILANA study evaluates the feasibility, acceptability and appropriateness of the implementation of CAB+RPV LA in clinics and community settings in the UK [8]. Surprisingly, only 27/114 people chose to receive injections in community settings; overall scores for feasibility, appropriateness and acceptability intervention measures (FIM/AIM/IAM) after 12 months of receiving the intramuscular injections at the hospital settings were higher (79.0–87.4%) than in the community settings (44.2–47.4%) [8]. Finally, at 7-month follow-up, preliminary data from the IMAdART study, which assessed treatment implementation and satisfaction across various care settings, including polyvalent day hospital (PDH) units and specialist care centers (SCC), indicated that treatment with CAB+RPV LA administered outside of HIV units was feasible. Participants reported high levels of treatment satisfaction, viral suppression rates remained robust, and discontinuations due to adverse events were infrequent [9].

To this point, there exists a notable absence of structured protocols or guidelines to assist healthcare providers in navigating the challenges and solutions associated with the administration of CAB+RPV LA within practical, real-world contexts outside of a traditional hospital setting. The implementation of complex chronic treatments in non-hospital settings presents both significant opportunities and challenges, according to the available scientific evidence [12,13]. However, assessing for organizational capacity and needs before and during the implementation of chronic care models, and understanding the perspectives of healthcare staff and stakeholders, remains essential [13,14].

Considering the scarce data on non-hospital-based administration of this antiretroviral combination, evaluating the implementation strategies is necessary to support successful implementation in alternative settings. This sub-study aims to analyze the implementation procedure of the non-hospital-based administration of CAB+RPV LA, specifically in terms of barriers and facilitators, from the point of view of staff participating in the HOLA study, a 12-month randomized, hybrid implementation–effectiveness, phase IV, double arm, open label, multicentric study to evaluate the acceptability, appropriateness, feasibility and satisfaction of out-of-hospital administration of CAB+RPV LA in virologically suppressed PWH [10]. This data will serve as the basis for its future implementation in other contexts, as well as to elaborate upon recommendations that broaden the reach of the intervention at a greater scale.

## 2. Methods

The current qualitative sub-study included staff participating in the HOLA study. The HOLA study is a 12-month randomized, hybrid implementation–effectiveness, phase IV, double arm, open label, multicentric study to evaluate the acceptability, appropriateness, feasibility and satisfaction of out-of-hospital administration of CAB+RPV LA in virologically suppressed PWH (NCT06185452) [15]. Non-hospital facilities included primary care centers, community centers and sexually transmitted infection (STI) clinics. Furthermore, the centers belonged to two different regions from Spain, including not only urban tertiary centers but also rural centers.

HOLA staff participants were recruited using purposive sampling by the principal investigators of each center that agreed to participate in this sub-study. Sample size was determined based on the principle of data saturation, defined as the point at which additional data no longer generates new theoretical insights or reveals new properties of conceptual categories [16].

This sub-study used a qualitative descriptive approach based on the Consolidated Framework for Implementation Research (CFIR). Data collection and analysis were conducted concurrently by two researchers (DH and JLM). Semi-structured individual online interviews, lasting 50–60 min, were conducted, and video-recorded to facilitate transcription and promote credibility. The interview guide was developed through a comprehensive process incorporating insights from existing literature and the research team. It addressed multiple topics related to the implementation of CAB+RPV LA. The guide was applied flexibly, adapting to the natural flow of conversation and the unique experiences shared by each participant. All participants signed Informed Consent and did not receive financial incentive for their participation. To avoid bias, all interviews were performed by the same researcher (DH). All interview transcripts were anonymous.

Data analysis used conventional content analysis, a technique commonly used in qualitative descriptive methods, which allows researchers to adapt their approach to study diverse healthcare phenomena [17] while remaining close to the data and the surface of words and events [18]. This approach focuses on providing a comprehensive summary of events or experiences in everyday language rather than deep interpretation [19]. Throughout the analysis, the team prioritized descriptive accuracy, aiming to present findings in participants’ own words and minimizing interpretive leaps. Representative quotations were carefully selected to illustrate each theme, thereby supporting transparency and trustworthiness in the reporting of results.

The analytic process began with repeated, attentive readings of the interview transcripts to achieve a thorough understanding of the data and to appreciate the full scope of participants’ perspectives. This phase of immersion allowed the research team to become closely acquainted with the content before proceeding to further analysis. Once familiarized, the transcripts were systematically divided into meaning units—distinct phrases or passages that each conveyed a single, relevant idea. This segmentation established a structured foundation for the next step, coding, ensuring that the analysis remained firmly anchored in participants’ own words and experiences. Coding was performed inductively, with categories and codes emerging directly from the data rather than being based on predefined frameworks. Each meaning unit was assigned a concise, action-oriented code that captured its essential idea or process, reflecting recurring topics and concepts within the dataset. The coding scheme was refined iteratively through continuous review and discussion among the research team, allowing for the emergence of new codes and consolidation of overlapping ones. To enhance credibility, two researchers independently coded the transcripts, and any discrepancies were resolved through consensus meetings, which promoted reflexivity and minimized individual bias. After coding, similar codes were grouped into broader categories and themes through a process of abstraction, moving from specific data segments to more general concepts that reflected patterns across the dataset.

The research team held multiple meetings to discuss and reach consensus on the findings, involving several rounds of review and discussion to ensure that identified themes accurately reflected the data and addressed the research questions [20]. To enhance credibility and trustworthiness, member checking was employed, involving sharing a summary of findings with a subset of participants to ensure the accurate representation of their experiences [21]. An audit trail was maintained throughout the analysis process, documenting all decisions and changes made [22]. Atlas.ti software version 22 was utilized to facilitate the systematic exploration and interpretation of the dataset.

The study adhered to the COREQ guidelines [23] and maintained rigor throughout the process [24].

## 3. Results

Thirteen professionals involved in the HOLA study participated in this sub-study. The total sample included both administrative roles (study coordinators) and healthcare roles (nurses, pharmacists and physicians), in three hospitals, one sexual health clinic and two primary healthcare centers. A brief sociodemographic description of the thirteen staff participants is presented in Table 1.

The analysis of the interviews conducted made it possible to identify five main categories with their respective subcategories (Table 2).

### 3.1. Operational and Infrastructure Adaptations

This category reflects the changes and adjustments needed in existing systems and infrastructures to accommodate the new treatment modality. Data showed that the introduction of the new treatment will necessitate substantial alterations to the physical infrastructure of the health centers. These modifications encompass the installation of dedicated equipment (e.g., a designated space for the administration of medication, secured and temperature-monitored space for the storage of medication) and the realignment of administrative areas to accommodate the revised patient flow and procedures. Space constraints also emerged as a recurrent issue in the interviews.

“It had to be set up in the outpatient day hospital. Two stretchers had to be placed in so that there would be two spaces where they could administer the medication.”

Nurse 1

Data showed concerns among professionals regarding the maintenance of drug stability during transport and storage, extending to all levels of the process, from the hospital to external facilities. The implementation of this system requires not only the acquisition of specialized equipment, such as refrigerators with temperature monitoring, but also the development of specific and strict coordination protocols and staff training.

“It’s really important to make sure we have a system that avoids breaking the cold chain. That’s been the most complicated part of logistics for us.”

Pharmacist

### 3.2. Integrated Management of Human and Organizational Resources

This category addresses the personnel required to implement the new injectable antiretroviral treatment, as well as the training needed to ensure effective and safe delivery. Furthermore, the category addresses issues related to the allocation and utilization of financial and material resources for implementation.

The data emerging from the study determines that an increase or a reorganization of staffing is necessary to accommodate the additional workload associated with implementing the new treatment in non-hospital facilities. It is not only nursing and pharmacy staff who require additional support, but also administrative and coordination roles.

This category also highlights the necessity of providing comprehensive training for staff, particularly in facilities where they lack familiarity with HIV treatment. Training encompasses not only the technical aspects of drug administration but also an understanding of the disease itself, and the needs of the patient.

The data also indicates a need to redefine and adapt the roles and responsibilities of staff involved in the implementation of the novel intervention. This includes the creation of new coordination roles and the adaptation of existing tasks.

“We could use one person or more staff to accommodate and prepare all the medication. Or we could extend the administration schedule and accommodate more patients by having more working hours or more people.”

Clinical trial coordinator 1

“The training for the nursing staff (in non-hospital facilities) should cover more than just drugs and administration. It should also include HIV, as specific knowledge is limited in the STI unit. And I imagine the same is true for Primary Healthcare Centers. Ultimately, to provide good patient care, we need to have training in the pathology itself.”

Nurse 4

The data shows a discrepancy between the financial assistance needed for the clinics and the actual constrained resources of the healthcare system. Similarly, some degree of optimism was expressed, albeit with reservations, about the financial support received or to be received. On the other hand, a concern about maintaining the independence of the medical process from the influence of the pharmaceutical industry was also expressed. In this context, the main positive influence from the industry was suggested to be the direct shipment of the medication from the pharmaceutical laboratory itself, which could solve many problems in maintaining the cold chain.

“I’d like to be able to do more things if I had more financial support. I’d be delighted, but I don’t know if I’m expecting it.”

Physician 1

“I hope there’ll be no outside help, and, in fact, I don’t want there to be outside help, which in any case would come from the industry, and that has to be another medical process, right? In that sense, the industry should keep a healthy distance.”

Physician 2

### 3.3. Need for Coordination and Follow-Up

Data indicated that the long-term implementation process requires significant adaptations in information systems and scheduling management. This will require the development of new protocols for patient follow-up, appointment scheduling and coordination between different departments and levels of care, including pharmacy, non-hospital facilities and the HIV unit. Flexibility in appointment scheduling was identified as a crucial factor, which requires changes not only to information systems but also to the organizational culture and the reality of staff and PWH, ensuring that both are supported in navigating the changes that come with more flexible appointment scheduling.

“The main change has been how we’ve organized the logistics with the day hospital. This means that when the patient comes for their injection, they already have it, and we can check that they’re complying with the admin side of things. We’ve also set up an alert system for when a patient doesn’t come in, so we can let them know and look for them.”

Physician 1

“I think it would be great to have a mobile app where people can communicate securely with the centers. They could also modify their appointments and have appointment reminders there to consult at any time.”

Clinical trials coordinator 2

Furthermore, data showed that it is essential to implement a structured approach to scheduling patient appointments within the designated administrative windows. It is therefore evident that there is a need for flexible and efficient scheduling systems that can accommodate patients’ needs while remaining within the required administration windows. There is a particular need to closely monitor patients’ adherence to treatment and any adverse effects that patients may experience, considering the patients’ target date and dosing window.

“The more we can adapt the schedules we offer them to fit in with their work life, the more we can help them, right? I think that’ll make it easier, because there are lots of people who, of course, if you limit yourself to Monday to Friday from 8 to 3… 70% of people work during those hours.”

Nurse 1

“The main issue is when a patient doesn’t show up at the administration desk. This happens a lot because there can be medical reasons for this, like developing resistance or making things worse.”

Physician 1

### 3.4. Professional Attitudes and Work Environment

This category outlines the significance of the human element in the change process, encompassing both the individual attitudes of professionals and the general work environment and institutional support. Readiness to change and staff motivation are identified as crucial factors for the effective implementation of the new treatment. The data illustrate a range of attitudes among professionals, from enthusiasm to resistance, with fear of change being the primary attitudinal challenge to overcome. This fear or resistance appears to be rooted in uncertainty and unfamiliarity with the new treatment.

“It’s the fear of the new. It’s as simple as that. It’s the fear of the unknown. That’s why so many centers have not wanted to participate or have not yet implemented it.”

Physician 2

Data emphasized that a participatory approach to implementing change is more effective than a top-down imposition from management. It was also highlighted that adaptability and a willingness to learn new skills are key attributes. Effective leadership and institutional support are identified as key factors in implementing the new treatment. Many participants emphasized the importance of support from senior management and a comprehensive approach involving multiple departments and levels of the organization. Data indicated that institutional support facilitates the logistical and bureaucratic aspects of implementation and sends a clear message about the importance of change. However, some healthcare professionals point out that institutional support does not necessarily imply additional resources.

“I think motivation is key. It’s down to the person in charge to get it going. With desire, you can achieve a lot and make things easy. But if you force it, it won’t work.”

Physician 3

“It’s important that the people running the service understand the case. The first thing we did was talk to the management to explain it. My hospital is very supportive of all-day hospital measures, and we haven’t had any problems with that. But it makes sense that, in the day hospital, the people running the day hospital have to approve it, which is what’s happened in our case.”

Physician 1

### 3.5. Patient Experience and Needs Perceived by Professionals

This category encompasses aspects pertaining to professionals’ perceptions of patients’ experiences with CAB+RPV LA. The participants’ experiences were found to be ambivalent, although the majority believed that the intervention would result in a better quality of life and greater accessibility due to the reduction in travel time, an advantage especially noted by those living far from the hospital. The ability to adapt administration schedules is also a key consideration. Flexibility is particularly important for patients who have other commitments that make it challenging to adhere to more rigid schedules. However, there are differences in people’s preferences, indicating that some individuals may favor the hospital setting due to the perception of enhanced safety and access to specialists.

“We’re saving them from having to make a 70 km one-way trip and then a 70 km return trip […] I’d say that most people prefer the hospital because it gives them more of a sense of security, and there are HIV doctors around.”

Physician 2

An evolution of the participant experience is identified, whereby they progressively adapt to the new treatment and its particularities. Adaptation to the new treatment and satisfaction is gradual as patients experience the benefits and become accustomed to the initial side effects.

“As the months go by, they are happier, especially because they don’t have to worry about the pill.”

Nurse 4

The data indicates that the implementation of treatment administration in non-hospital settings could contribute to the reduction of stigma associated with HIV. However, confidentiality concerns persist, mainly related to the potential loss of anonymity when attending primary healthcare centers, where it is much easier to encounter someone known, especially in non-urban settings.

“If you normalise it a little bit more, as the person who comes to get any other intramuscular treatment, you can also help to destigmatize it in the face of healthcare personnel, where stigma also exists.”

Nurse 4

“Some patients don’t want to know anything about their health centre, but it’s not because of anything the centre does, it’s just that the quotas are based on geography.” “These are the streets that are right next to yours, in your neighbourhood. You don’t want anyone to know that you go to the health centre every two months to get a shot. So, there are people who prefer the anonymity of the hospital, and there are people who prefer to be pricked at their health centre.”

Physician 2

## 4. Discussion

Our findings highlighted key factors influencing the implementation of CAB+RPV LA outside hospital settings as perceived by professionals, including operational and infrastructure adaptations, effective resource management, coordination and follow-up, professional attitudes and work environment and patient needs. These factors collectively provide a framework for understanding the challenges and opportunities of transitioning this intervention to non-hospital contexts.

### 4.1. Summary of Considerations for Implementation

Similarly to published data in hospital settings [5,25,26,27], our study highlights the operational and infrastructure adaptations required for the successful implementation of CAB+RPV LA, similar to what researchers from the Phase III CUSTOMIZE trial have previously indicated [27]. Our qualitative data indicated that physical space adaptations in health centers are necessary to accommodate the new treatment, highlighting the need for careful planning and resource allocation, especially when considering the implementation in the long term, due to the increase in patient volume and conflicting resources. Potential solutions identified include repurposing existing areas—such as dedicated consultation rooms, vaccination areas, or spaces currently used for other injectable therapies—while ensuring that patient privacy is maintained. Sustainable financial planning is also necessary to secure long-term funding and resource allocation, which could be achieved through public health initiatives or collaborations with the pharmaceutical industry.

Maintaining the cold chain and logistics of the medication emerged as a critical challenge. Staff expressed concerns about ensuring the integrity of the medication during transport and storage, needing specialized equipment and strict protocols. This data is aligned with general views of staff in other HIV units [28]. Implementing dedicated refrigeration units with continuous temperature monitoring is critical, as well as establishing robust coordination protocols for timely medication delivery. The establishment of standardized protocols for drug handling, storage and administration is also crucial to ensure consistency and safety. To aid in the cold chain maintenance, the possibility of a direct supply from the pharmaceutical company may simplify the process and guarantee the delivery of medication in the appropriate conditions. However, there is no consensus, particularly from the point of view of hospital-centralized roles such as the pharmacy or HIV specialists.

Another crucial aspect for the successful implementation of CAB+RPV LA is human resources and training, as supported by multiple studies [5,25,29]. Difficulties in care coordination and challenges in monitoring and follow-up outside the hospital environment need to be addressed appropriately [30], including by improving the training of primary care providers [12]. As aligned with prior CAB+RPV LA implementation studies, our data indicate that there may be a need for additional personnel, particularly nurses, to manage the increased workload. Our data also indicated that redefining roles and responsibilities within the team is necessary to ensure effective coordination and follow-up. The need for a clinical coordinator was also identified as crucial. This figure would ensure continuing quality care (i.e., coordination of agendas, management of medication stocks and coordination with pharmacy, administration conditions, management of treatment windows, or management of adverse events). In addition, training was considered essential, especially for staff unfamiliar with HIV treatment, covering both technical aspects of medication administration and broader knowledge about HIV infection, patient communication, and adherence support. Training sessions should be conducted prior to the implementation phase to ensure that all professionals involved are adequately prepared.

Moreover, our study identifies the need for systematic logistics considering the frequency of shipments and allowance of stock to permit flexible agendas, as well as reminders and alerts to assure the adherence to treatment. These findings complement data from previous implementation studies, supporting efforts in staff training [4] and the designation of specific roles for coordination and follow-up systems [27]. Fostering interdepartmental collaboration between HIV units, pharmacies and external healthcare centers would help to create a seamless care pathway, ensuring that patients receive their treatment in a timely and efficient manner. Improving appointment scheduling systems and incorporating flexible rescheduling options within the administration window, as well as the implementation of proactive follow-up mechanisms, such as alert systems for missed visits, would further support patient retention and treatment success. In this regard, there are some tools already available specifically for this purpose that can help.

As shown, professional attitudes and the work climate play an essential role in the implementation process. Furthermore, the literature highlights the importance of factors such as organizational culture, structural characteristics of health institutions, networks and communication, implementation climate and readiness, as well as the attitudes and beliefs of healthcare providers [14]. Some professionals were enthusiastic about the new treatment, while others exhibited resistance due to fear of change and unfamiliarity with the new procedures. Effective leadership and institutional support are critical in overcoming these challenges, promoting a participatory approach to change rather than top-down imposition; this includes engaging in decision-making processes and advocating for the new model of care, transparent communication and the involvement of healthcare staff in planning and implementation. These actions can mitigate resistance and improve acceptance, based on our findings. It was clear in our interviews that the key figure in the implementation was the nurse, and nursing team, who oftentimes were engaged in administrative tasks and coordination. Nevertheless, considering the potential resource limitations, most of the participants interviewed agreed that with willingness from all personnel involved, implementation was possible in all contexts.

### 4.2. Patient-Centered Considerations

Finally, considering patients’ needs, some professionals believe that the decentralization of treatment might help in normalizing the condition [31]. It should be noted that HIV stigma is a complex issue [32] and has a detrimental impact on a variety of health-related outcomes [33]. In line with this, community services provide a safe environment for PWH communities, which might make these strategies particularly successful [34], likely translating into higher patient engagement, treatment adherence and psychosocial improvements. However, internalized stigma prevented some patients from participating in the HOLA study due to feeling uncomfortable being attended outside the familiar and supportive environment provided by the hospital, as was indicated by some interviewed professionals. Therefore, there is also a clear importance of staff training in the psychological aspects accompanying the diagnosis, as well as a good professional–patient bond and frequent communication. Our data was aligned with qualitative outcomes of the ILANA study, where HIV stigma and confidentiality emerged as obstacles for switching to a new setting [8]. With regard to this, it is important to note that contrary to the HOLA study, in the ILANA study, receiving CAB+RPV LA in the clinic or a community setting was optional after six months of receiving treatment, rather than something participants were randomized to. As for the IMAdART study, preliminary data indicated that 65.5% of participants expressed some degree of concern regarding the disclosure of their HIV status in the last 6 months [10]. At the 7-month follow-up, improvements from baseline in terms of psychosocial events related to the ART therapy became significantly evident, and concerns regarding disclosure of their HIV status decreased by 22.6 percentage points [9]. In the GLACIER study, study participants reported benefits in terms of privacy and reduced stigma [4]. It must be noted that in our sub-study, direct measures of stigma were not performed in PWH, and this point is solely based on the opinion of the professionals. Nevertheless, other studies have identified a minimized effect of HIV-related stigma in people participating in interventions involving the implementation of CAB+RPV LA outside of the hospital setting in the US (GLACIER, in press).

### 4.3. Limitations and Next Steps

The study has a main limitation, which is the limited number of participating centers. This is due to data saturation being based on general results and not on the number of participants from each center. Despite this, all different types of settings and roles were represented. Additionally, differences regarding the healthcare systems between regions or countries imply difficulties in generalizing the results, which could limit their applicability to other contexts where resources, infrastructures or regulatory requirements differ. It is essential to emphasize that the solutions provided in this paper are not universally applicable, as each healthcare center has its own organizational structure, resources, and patient population characteristics. Thus, any adaptation must be tailored to the specific context in which it is implemented. This study included practitioners from urban and rural centers and different types of clinics (hospital, primary healthcare, community and STI clinics) from different regions of Spain to expose different settings and potential barriers and minimize the difficulties in translating our results to other centers.

In conclusion, this study provides a detailed analysis of the practical challenges and solutions associated with implementing CAB+RPV LA outside the hospital setting. While there are some structural changes which might be challenging depending on the existing infrastructure of each alternative outpatient center, the data presented in this manuscript provides a blueprint of the step-by-step barriers and facilitators that might be encountered, and has the potential to help in the resolution process. The successful implementation of CAB+RPV LA requires comprehensive operational and infrastructure adaptations, adequate human resources and training and a supportive professional environment. Effective leadership and institutional support are crucial for overcoming resistance to change and ensuring the sustainability of the new treatment model. PWH needs must be considered throughout the externalization process. Future efforts should focus on addressing the identified challenges and exploring strategies to enhance the implementation process, not only for the administration of CAB+RPV LA but also for other potential LA therapies that may emerge in the near future.

## Figures and Tables

**Table 1:** Sociodemographic characteristics of the staff participants.

Gender	
Man Woman N/A	6 6 1
Age	44.00 (±10.86) *
Region	
Barcelona Malaga	9 4
Profession	
Trial coordinator Pharmacist Nurse Physician	3 1 4 5
Years of professional experience	16.23 (±10.03) *

* Mean (standard deviation).

**Table 2:** Categories and subcategories of the interview.

Category	Subcategory
Operational and infrastructure adaptations	▪ Adaptation of physical spaces and equipment ▪ Cold chain management and drug logistics
Integrated management of human and organizational resources	▪ Strategic resource planning ▪ Human capital development
Need for coordination and follow-up	▪ Information systems and agenda management ▪ Reminder and alert systems ▪ Appointment management and consideration of administration windows ▪ Monitoring of adherence and adverse effects
Professional attitudes and work environment	▪ Willingness to change and motivation of staff and group ▪ Leadership and institutional support
Patient experience and patients’ needs perceived by professionals	▪ Accessibility and patient preferences ▪ Adapting to the new model of care and treatment ▪ Psychosocial aspects (confidentiality, stigma vs. anonymity, normalization)

## Data Availability

Individual personal data are protected and not available due to applicable data privacy legislation: LOPD, The Organic Law 3/2018 of 5 December on the Protection of Personal Data and the Guarantee of Digital Rights complementary to the Regulation (EU) 2016/679 of the European Parliament and of the Council of 27 April 2016, on the protection of natural persons with regard to the processing of personal data and on the free movement of such data. The deidentified datasets used/or analyzed during the current study might be available from the corresponding author on reasonable request.

## Abbreviations

LA	Long-acting
HIV	Human immunodeficiency virus
CAB+RPV	Cabotegravir and rilpivirine
CFIR	Consolidated Framework for Implementation Research
PWH	People with HIV
AEs	Adverse events
